# The relationship between oxidative balance score, depression, and survival among adult cancer survivors in the United States

**DOI:** 10.3389/fnut.2025.1622588

**Published:** 2025-07-16

**Authors:** Qu Zhang, Yemei Wu, Qianyu Fan, Wenxi Zhou, Min Liu

**Affiliations:** ^1^Department of Radiation Oncology, The Second Affiliated Hospital of Anhui Medical University, Hefei, Anhui, China; ^2^Department of Radiotherapy Center, Hubei Cancer Hospital, Tongji Medical College, Huazhong University of Science and Technology, Wuhan, China; ^3^Hubei Cancer Hospital, Tongji Medical College, Huazhong University of Science and Technology, Wuhan, China; ^4^Department of Thoracic Oncology, Hubei Cancer Hospital, Tongji Medical College, Huazhong University of Science and Technology, Wuhan, China; ^5^National Key Clinical Specialty Discipline Construction Program, Hubei Provincial Clinical Research Center for Breast Cancer, Wuhan Clinical Research Center for Breast Cancer, Breast Cancer Center, Hubei Cancer Hospital, Tongji Medical College, Huazhong University of Science and Technology, Wuhan, Hubei, China

**Keywords:** oxidative balance score, depression, cancer survivors, mortality, NHANES

## Abstract

**Background:**

Depression and oxidative balance score (OBS) are linked to disease risk, yet their combined effects on cancer survival remain unclear. This study assessed OBS, depression, and mortality in cancer survivors.

**Methods:**

Utilizing a prospective, population-based cohort design, this analysis enrolled 1,455 adult cancer survivors (age ≥20 years) through the National Health and Nutrition Examination Survey (NHANES) from 1999 to 2018. The OBS was related to diet and exercise, and depression was self-reported. Depressive symptomatology was measured using the established Patient Health Questionnaire-9 (PHQ-9) self-report questionnaire. Depression was defined as a total PHQ-9 score > 4, indicating the presence of depressive symptoms. A score ≤ 4 was considered to indicate no depression. Mortality outcomes (all-cause, cancer-specific, non-cancer) were tracked via the National Death Index through 2019. Cox models adjusted for demographics, socioeconomic status, and comorbidities.

**Results:**

Over 80–90 months, 329 deaths occurred (102 cancer-related). Higher OBS predicted reduced mortality (per-unit HR = 0.94, 95% CI: 0.90–0.98). In OBS tertiles, Tertile 3 vs. Tertile 1 showed HR = 0.30 (95% CI: 0.14–0.63) for cancer mortality. Depression alone had no mortality association (HR = 1.24, 95% CI: 0.49–3.18). However, within the highest OBS tertile, depressed patients exhibited lower cancer mortality (HR = 0.18, 95% CI: 0.05–0.71) versus non-depressed counterparts.

**Conclusion:**

Elevated OBS is protective in cancer survivors. Depression may paradoxically reduce mortality risk in high-OBS subgroups, suggesting nutrition-psychology interactions.

## Background

The pathophysiological synergy between elevated redox imbalance and clinically significant depression potentiates the development of multimorbidity and accelerates mortality trajectories across disease states. Nevertheless, their specific influence on mortality outcomes among cancer survivors is yet to be fully elucidated.

Advances in diagnostic technologies and refinement of therapeutic approaches have led to a marked improvement in cancer patient survival rates, contributing to a steady rise in the global population of cancer survivors. Projections indicate that by 2025, approximately 2,041,910 new cancer cases will be diagnosed, with 618,120 cancer-related deaths expected in the United States (1). Nevertheless, long-term health challenges have become the main problem faced by cancer survivors and have a profound impact on their survival outcomes. The survival rate of cancer survivors is not only affected by the primary disease but also restricted by multiple metabolic, psychological, and lifestyle factors (2).

Oxidative stress plays a crucial role in the occurrence, recurrence, and progression (3). It refers to cellular and tissue damage caused by an imbalance between reactive oxygen species and antioxidants (4). This imbalance not only promotes DNA damage and gene mutations but also activates cancer-related inflammatory pathways, accelerating cancer recurrence and metastasis (5). In addition, cancer survivors often have elevated levels of oxidative stress, which may stem from long-term side effects of treatments such as chemotherapy and radiotherapy (6). Oxidative stress is widely recognized as a critical pathological driver of cancer development and progression (7). The OBS is an index that comprehensively evaluates an individual’s oxidative stress status, calculated based on the intake of dietary antioxidants (such as vitamins C and E and carotenoids) and oxidant exposure (such as smoking and lipid peroxidation) (8). OBS can reflect an individual’s oxidative stress exposure level and health status, providing a new perspective for assessing the long-term survival risk of cancer survivors.

The oxidative balance system is modulated by a variety of dietary components with distinct biochemical roles. Pro-oxidant factors such as saturated fats, heme iron, alcohol, and smoking-related metabolites (e.g., cotinine) contribute to excessive reactive oxygen species (ROS) generation via mitochondrial dysfunction, Fenton reactions, and cytochrome P450 induction. For instance, dietary iron catalyzes ROS formation through the Haber–Weiss cycle, thereby promoting lipid peroxidation and DNA strand breaks. Conversely, antioxidants including vitamins C and E, carotenoids, flavonoids, selenium, and zinc scavenge ROS or enhance endogenous defense mechanisms like glutathione peroxidase and superoxide dismutase. Vitamin C functions as a water-soluble electron donor that neutralizes hydroxyl radicals, while vitamin E protects cell membranes from lipid peroxidation. These nutrients are predominantly sourced from fruits (e.g., citrus, berries), vegetables (e.g., spinach, broccoli), nuts, whole grains, and seafood. The balance between these opposing nutrient classes forms the basis of the OBS, a composite metric designed to reflect an individual’s overall oxidative stress burden and redox status.

Simultaneously, depression ranks as one of the most prevalent psychological disorders among cancer survivors, with its prevalence notably higher than in the general population (9). Depression has an adverse impact on the prognosis of patients with cancer through hypothalamic–pituitary–adrenal (HPA) axis dysfunction, increased levels of inflammatory factors (such as interleukin-6 and C-reactive protein), and behavioral risk factors (such as smoking and unhealthy diet) (10). Previous studies demonstrated a bidirectional interaction between depression and oxidative stress. Depression may exacerbate oxidative stress, which in turn may exacerbate depressive symptoms (6). However, research on the interactive effects of OBS and depression on the long-term survival rate of cancer survivors remains limited.

The long-term survival of cancer survivors is affected by multiple factors, among which oxidative stress and mental health problems are particularly important. The OBS has been widely used to evaluate the role of oxidative stress in chronic diseases such as cardiovascular diseases (11) and metabolic syndrome (8); however, its application in the context of cancer survivors is still in its infancy (12). Similarly, although depression is considered an important factor affecting the survival of patients with cancer (13), current research mainly focuses on the relationship between depression, cancer recurrence, and quality of life. There is also insufficient research on the combined effects of oxidative stress and survival rate (14).

This study aimed to fill the research gap in this field and explore the relationship between OBS, depression, and survival of adult cancer survivors in the United States. The specific objectives were (1) to assess the independent effects of OBS and depression on the survival rate of cancer survivors and (2) to analyze the interaction between OBS and depression and its impact on survival outcomes. We hypothesized that a higher OBS is associated with a better survival rate, whereas depression is associated with a worse survival outcome. In addition, the coexistence of depression and low OBS may have a synergistic negative impact on survival.

Through a comprehensive analysis of oxidative stress and depression, this study not only provides a new perspective on the health management of cancer survivors but also provides a scientific basis for the development of personalized intervention strategies (such as antioxidant supplementation and psychotherapy), thereby improving the long-term survival and quality of life of cancer survivors.

## Methods

### Study population

This study analyzed nationally representative data from the National Health and Nutrition Examination Survey (NHANES), an ongoing biennial surveillance program assessing population health metrics in the U. S. NHANES employs multistage stratified probability sampling across geographic units (county/census tract/household), oversampling specific subgroups to ensure demographic representativeness. Complex weighting adjustments accounted for oversampling, nonresponse, and poststratification according to NHANES analytic guidelines.

As part of the NHANES, the protocols employed in this study were approved by the Ethics Review Committee of the National Center for Health Statistics and the Centers for Disease Control and Prevention. Written informed consent was obtained from all the participants prior to their inclusion in the study.

**Figure 1:**
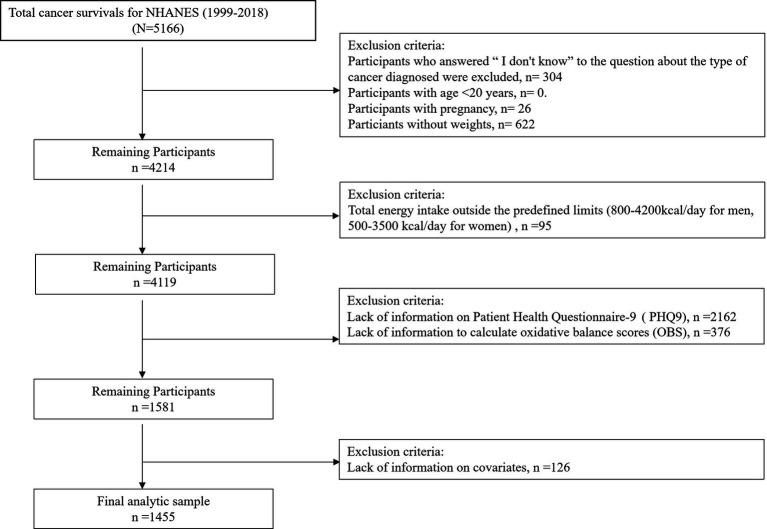
Flow chart for the study population.

Data collection involved structured interviews, clinical assessments, and biomarker analyses performed at NHANES mobile examination centers (MECs). This retrospective cohort analysis included 1,455 adult cancer survivors (age ≥20 years) participating in NHANES cycles from 1999 to 2018. Participant stratification is detailed in Figure 1, which delineates the eligibility screening protocol. Since the data excludes personally identifiable information, neither informed consent nor Institutional Review Board approval was required.

### Cancer assessment

Cancer-related metadata (tumor multiplicity, age of onset, ≤3 primary diagnoses) were ascertained via structured self-report instruments. Survivorship status was confirmed through physician-verified malignancy history using the query: “Have you ever received a cancer diagnosis from a healthcare provider?” Subsequent items captured histologic subtypes and initial diagnosis age. Cancer duration was computed as current age minus first-diagnosis age, excluding non-malignant neoplasms through systematic filtering.

### Calculation of the OBS

The OBS construction methodology follows well-established protocols (8), integrating 16 dietary micronutrients and four lifestyle parameters categorized as pro-oxidants (total fat, iron, alcohol, BMI, cotinine) and antioxidants (fiber, *β*-carotene, B vitamins, vitamins C/E, calcium, magnesium, zinc, copper, selenium) along with physical activity. Dietary components were quantified using averaged 24-h recall data (supplements excluded per protocol). Physical activity intensity was assessed via MET-hour calculations, while serum cotinine levels quantified smoking exposure. Antioxidant tertiles were scored 0–2 (low-high), with pro-oxidants inversely scored. Comprehensive OBS component specifications and tertile stratification are detailed in Supplementary Table S1, respectively.

### Assessment of depression

Depressive symptomatology in cancer survivors was assessed using the validated Patient Health Questionnaire-9 (PHQ-9), which evaluated symptom frequency over the preceding 14 days. This instrument has established psychometric properties in oncological populations (15, 16). The nine-item scale quantifies manifestations including anhedonia, depressed affect, sleep dysfunction, fatigue, appetite changes, guilt, concentration impairment, psychomotor alterations, and suicidal ideation. Each item is rated 0 (never) to 3 (daily), generating a total score of 0–27 that correlates with symptom severity. Using established thresholds (17), scores were dichotomized into non-clinical (0–4) versus clinically significant (≥5) depressive states.

### Mortality determination

The investigation centered on three mortality endpoints: all-cause, cancer-specific, and non-cancer mortality. Vital status was ascertained through probabilistic linkage of NHANES mortality records (updated through December 2019) with the National Death Index. Mortality causality was classified per ICD-11 guidelines: neoplastic deaths (C00-C97) versus non-cancer etiologies. Follow-up duration was quantified as the number of months from baseline assessment to death or study conclusion (December 31, 2019) for survivors.

### Covariates

Covariates were selected based on established theoretical frameworks and empirical evidence. Sociodemographic variables (age, sex, race/ethnicity, education, marital status, income-poverty ratio, employment) were collected via standardized questionnaires. Racial/ethnic categorization followed National Center for Health Statistics criteria: Mexican American, Other Hispanic, non-Hispanic White, non-Hispanic Black, and other/multiracial. Educational attainment was categorized into three levels: less than high school, high school, college or above. Marital status comprised four classifications: married, unmarried, cohabiting, other (widowed/divorced/separated). Family income-to-poverty ratios were stratified into three tiers (<1.30, 1.31–3.50, > 3.50) (18). Comorbidities including diabetes, hyperlipidemia, and cardiovascular disease were also incorporated.

Data on antidepressant usage were extracted from the NHANES prescription information file. Information regarding medication use was collected during in-home interviews, in which participants were queried about their drug consumption over the preceding 30 days. Those who responded affirmatively were asked to present their medication containers; otherwise, they were requested to report the names of the drugs verbally. Prescription drug data were processed and categorized using the Lexicon Plus database, with “Psychotherapeutic drugs” as the primary classification and “Antidepressants” as the secondary category. In this study, the use of one or more antidepressant medications within the past 30 days was defined as antidepressant use.

### Statistical analysis

All statistical analyses accounted for the complex sampling design of NHANES to ensure nationally representative and unbiased estimates. Sample weights, strata, and primary sampling units were incorporated in accordance with the NHANES analytic guidelines to adjust for differential probabilities of selection, nonresponse, and oversampling of specific subpopulations (19).

Analyses were conducted using R version 4.3.0. A two-sided *p*-value < 0.05 was considered statistically significant. Survey-weighted Cox proportional hazards regression models were used to estimate hazard ratios (HRs) and 95% confidence intervals (CIs) for all-cause, cancer-specific, and non-cancer mortality.

Baseline characteristics were stratified by OBS tertiles and depression status. Multivariable Cox models were constructed to evaluate the independent and joint effects of OBS and depression on mortality outcomes. Participants were categorized into subgroups based on OBS and PHQ-9-defined depression status to assess interaction effects.

Two hierarchical multivariable models were developed: Model 1 adjusted for demographic and socioeconomic variables, including age, sex, race/ethnicity, education level, marital status, and family income-to-poverty ratio (PIR). Model 2 included all variables in Model 1 and further adjusted for NHANES cycle, cancer type, years since initial cancer diagnosis, comorbidities (diabetes, hyperlipidemia, cardiovascular disease), total energy and caffeine intake, physical activity level, and antidepressant use.

This tiered modeling approach allowed assessment of effect stability across varying levels of covariate control and minimized residual confounding.

Subgroup analyses were performed using stratified Cox models to evaluate the robustness of associations across age groups, sex, education, marital status, PIR categories, comorbidity burden, and antidepressant use. Interaction terms were evaluated by likelihood ratio tests comparing models with and without the interaction term between OBS and depression. Model coefficients were tested using Wald chi-square statistics.

## Results

### Basic characteristics of the study population

Figure 1 illustrates the flowchart of participant inclusion and exclusion across NHANES cycles, yielding the final analytic sample (*n* = 1,455).

A total of 1,455 cancer survivors with a mean age of 62.33 years, comprising 851 females (58%) and 604 males (42%). Significant variations were observed in the family income-to-poverty ratio and energy intake (*p* < 0.0001), with the highest values in tertile three and the lowest in tertile one. Additionally, significant differences were noted in marital status, education level, and racial distribution across groups (*p* < 0.05) (Table 1). The detailed baseline characteristics of the study population are provided in Supplementary Tables S2, S3.

**Table 1:** The baseline characteristics by tertiles of the OBS: National Health and Nutrition Examination Survey 1999–2018 (NHANES 1999-2018)^a^.

Characteristics	All	Tertile 1	Tertile 2	Tertile 3	*P*
Age	62.33(0.58)	60.94(1.11)	62.46(1.06)	63.18(0.88)	0.0896
Family poverty income ratio	3.19(0.07)	2.55(0.11)	3.3(0.12)	3.54(0.11)	<0.0001
Energy intakes	1915.5(29.23)	1501.47(34.57)	1839.72(40.42)	2267.81(40.35)	<0.0001
Caffeine intakes	176.32(6.09)	180.48(14.81)	170.52(9.87)	178.42(8.54)	0.6680
Marital status					0.0001
Married	846(64.07)	243(52.81)	302(69.39)	301(67.33)	
Unmarried	609(35.93)	221(47.19)	182(30.61)	206(32.67)	
Educational attainment					<0.0001
Less than high school	273(9.98)	138(17.81)	84(9.22)	51(5.2)	
High school or equivalent	343(21.46)	128(27.84)	101(19.57)	114(18.65)	
College or above	839(68.56)	198(54.34)	299(71.21)	342(76.15)	
Race					0.0187
Non-Hispanic White	1,020(86.39)	292(81.8)	340(85.22)	388(90.58)	
Non-Hispanic Black	190(4.78)	83(6.89)	57(4.97)	50(3.16)	
Mexican American	90(2.33)	38(3.29)	33(2.48)	19(1.53)	
Other Race-Including Multi-Racial	155(6.5)	51(8.02)	54(7.33)	50(4.73)	
History of comorbidities, no (n, %)					0.0673
No	433(35.96)	108(29.4)	140(34.93)	185(41.39)	
Yes	1,022(64.04)	356(70.6)	344(65.07)	322(58.61)	
Antidepressant (n, %)					0.1923
No	1,359 (91.19)	423 (88.41)	460 (90.82)	476 (93.44)	
Yes	96 (8.81)	41 (11.59)	24 (9.18)	31 (6.57)	

a All estimates accounted for complex survey designs in NHANES. Values were mean ± standard error for continuous variables and numbers (percentages) for categorical variables.

OBS, oxidative balance scores.

**Figure 2:**
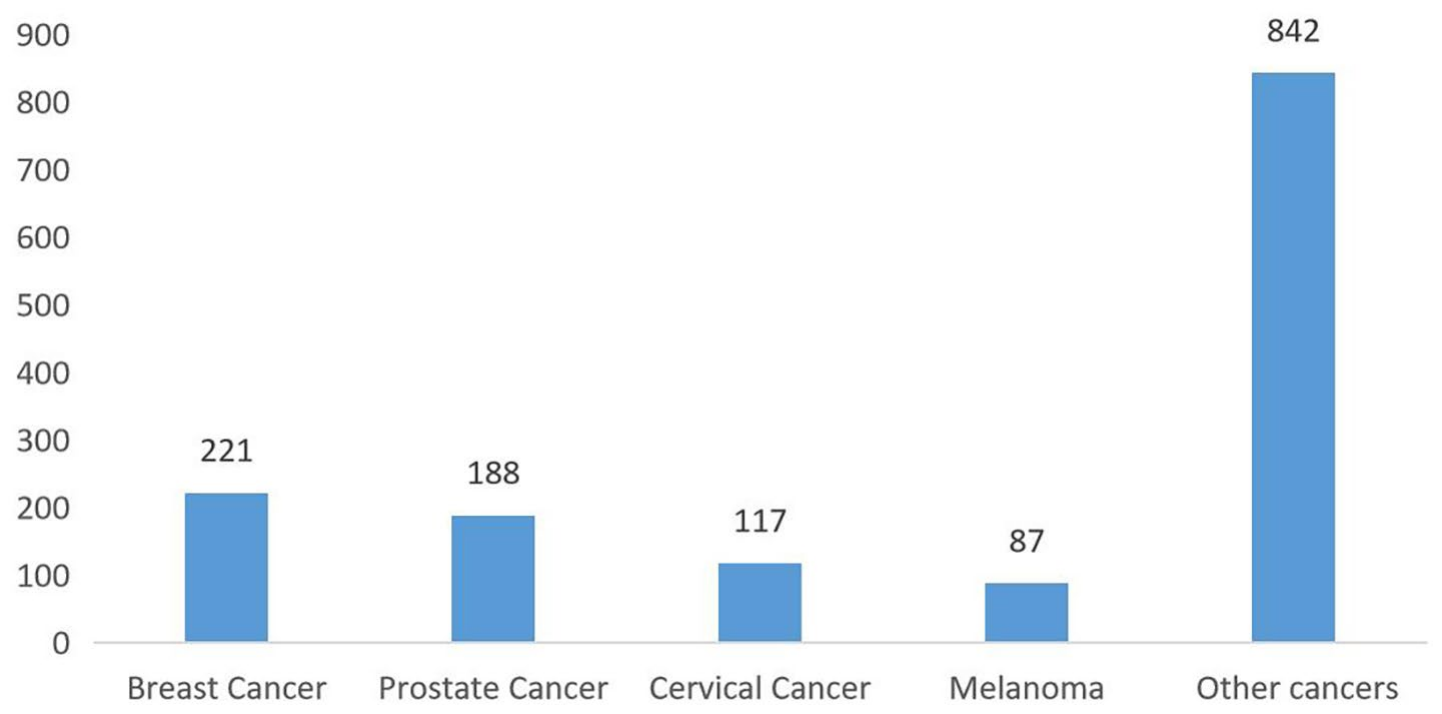
Number of different cancers in the total population.

Figure 2 shows the cancer type distribution among survivors, with breast (15%), prostate (13%), and cervical cancers (8%) being the most common. The distribution of cancer types was as follows: 221 cases of breast cancer (15%), 188 of prostate cancer (13%), 117 of cervical cancer (8%), 87 of melanoma (6%), and 842 of other cancer (58%) (Figure 2). The distribution of cancer types according to sex is illustrated in Supplementary Figures S1, S2.

**Figure 3:**
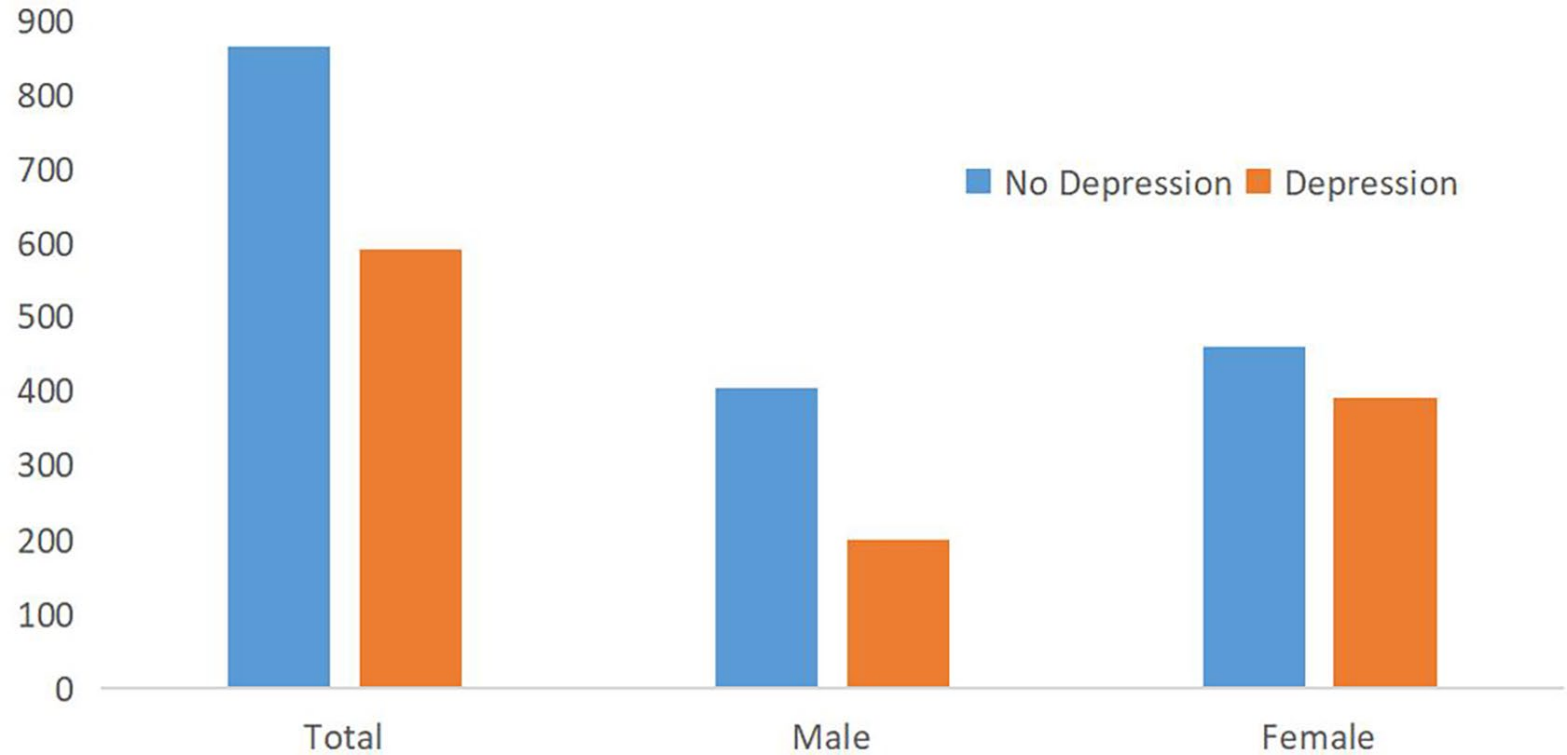
Number of depression and no depression in males and females.

Figure 3 compares depression prevalence by sex, highlighting a higher proportion of depressive symptoms among females. Regarding depression status, according to the PHQ-9 score, 864 participants did not have depression (59.38%), and 591 had depression (40.62%). There were 200 males (33.84%) and 391 females (65.16%) with depression (Figure 3).

### Independent relationship between OBS and survival

Multivariable-adjusted Cox regression, accounting for demographic factors (age/sex/race), socioeconomic status (education/marital status/income-poverty ratio), and nutritional variables (energy/caffeine intake), demonstrated a significant OBS-survival association in cancer survivors (Table 2).

**Table 2:** Association of OBS with all-cause, cancer, and noncancer mortality among US cancer survivors aged 20 years or older, National Health and Nutrition Examination Survey 1999–2018.

Mortality outcome	Death/no	MV model 1^a^	MV model 2^b^
All cause			
OBS			
Tertile 1	118/464	1.00(Reference)	1.00(Reference)
Tertile 2	105/484	0.74(0.49–1.12)	0.92(0.63–1.36)
Tertile 3	106/507	0.63(0.44–0.92)	0.79(0.56–1.12)
Per one point	NA	0.97(0.96–0.99)	0.99(0.97–1.01)
P for trend	NA	0.0178	0.1775
Cancer			
OBS			
Tertile 1	47/464	1.00(Reference)	1.00(Reference)
Tertile 2	26/484	0.32(0.15–0.69)	0.41(0.20–0.84)
Tertile 3	29/507	0.30(0.14–0.63)	0.38(0.20–0.76)
Per one point	NA	0.94(0.90–0.98)	0.96(0.92–0.99)
P for trend	NA	0.0032	0.0072
Noncancer			
OBS			
Tertile 1	71/464	1.00(Reference)	1.00(Reference)
Tertile 2	79/484	1.10(0.70–1.75)	1.34(0.87–2.07)
Tertile 3	77/507	0.91(0.63–1.31)	1.11(0.76–1.62)
Per one point	NA	0.99(0.97–1.01)	1.00(0.98–1.02)
P for trend	NA	0.5274	0.6882

a Adjusted for age, sex.

b Adjusted for age, sex, race (Non-Hispanic White, Non-Hispanic Black, Mexican American, others), educational attainment (Less than high school, High school or equivalent, College or above), marital status (Married, Unmarried), Family poverty income ratio, Energy intakes, Caffeine intakes.

OBS, oxidative balance scores.

For all-cause mortality, in MV model 1, as the OBS tertile increased, the mortality risk decreased. Specifically, when comparing tertile three with tertile one, the Hazard Ratio (HR) was 0.63 (95% CI: 0.44–0.92). The HR for a one—point increase was 0.97 (95% CI: 0.96–0.99; *p* = 0.0178). However, in MV model 2, this trend weakened, with the HR for a one - point increase being 0.99 (95% CI: 0.97–1.01; *p* = 0.1775).

Regarding cancer mortality, in MV models 1 and 2, the higher the OBS tertile, the lower the cancer mortality risk (for example, in MV model 2, tertile 3 vs. tertile 1, HR = 0.38, 95% CI: 0.20–0.76). The HR per one-point increase was 0.94 (95% CI: 0.90–0.98) and 0.96 (95% CI: 0.92–0.99), respectively, with *p*-values of 0.0032 and 0.0072.

Regarding noncancer-related mortality, there was no significant linear association with OBS (the trend *p*-values in MV models 1 and 2 were 0.5274 and 0.6882, respectively).

The results indicated that patients with higher OBS had a higher survival advantage owing to their lower oxidative stress state.

### Independent relationship between depression and survival

Multivariable Cox regression adjusted for demographic factors (age, sex, race/ethnicity), socioeconomic status (education, marital status, income-poverty ratio), and nutritional variables (energy/caffeine intake) demonstrated no significant association between depression and survival outcomes in cancer survivors (Table 3).

**Table 3:** Association of depression status with all-cause, cancer, and noncancer mortality among US cancer survivors aged 20 years or older, National Health and Nutrition Examination Survey 1999–2018.

Mortality outcome	Death/No	MV model 1^a^	MV model 2^b^
All cause			
No	286/1248	1.00(Reference)	1.00(Reference)
Yes	43/207	1.75(1.17–2.62)	1.23(0.84–1.82)
Cancer			
No	84/1248	1.00(Reference)	1.00(Reference)
Yes	18/207	2.04(0.84–4.95)	1.24(0.49–3.18)
Noncancer			
No	202/1248	1.00(Reference)	1.00(Reference)
Yes	25/207	1.58(0.96–2.60)	1.22(0.76–1.95)

a Adjusted for age, sex.

b Adjusted for age, sex, race (Non-Hispanic White, Non-Hispanic Black, Mexican American, others), educational attainment (Less than high school, High school or equivalent, College or above), marital status (Married, Unmarried), Family poverty income ratio, Energy intakes, Caffeine intakes.

For all-cause mortality, multivariable-adjusted Model 1 demonstrated elevated risk among depressed versus non-depressed patients (HR = 1.75, 95%CI 1.17–2.62), whereas Model 2 showed attenuated non-significant association (HR = 1.23, 95%CI 0.84–1.82).

Regarding cancer mortality, in MV model 1, the cancer death risk of patients with depression was higher than that of those without depression (HR = 2.04, 95% CI: 0.84–4.95). Moreover, the difference was not statistically significant in MV model 2 (HR = 1.24, 95% CI: 0.49–3.18).

Regarding noncancer mortality, in MV models 1 and 2, although there were differences in noncancer mortality between patients with and without depression, the difference was not significant in MV model 2.

### Interaction between OBS and depression

The Cox proportional hazards model, adjusted for demographic characteristics (age, sex, race/ethnicity), socioeconomic factors (education, marital status, income-poverty ratio), and dietary patterns (energy/caffeine intake), revealed significant associations among OBS, depression, and cancer survivorship outcomes (Table 4).

**Table 4:** Joint association of OBS and depression status with all-cause, cancer, and noncancer mortality among US cancer survivors aged 20 years or older, National Health and Nutrition Examination Survey 1999–2018.

Mortality outcome	Depression	Death/no	MV model 1^a^	MV model 2^b^
All cause				
Tertile 1	No	96/360	1.00(Reference)	1.00(Reference)
Tertile 1	Yes	22/104	1.97(0.95–4.07)	1.32(0.62–2.83)
Tertile 2	No	94/432	0.78(0.50–1.23)	0.95(0.61–1.46)
Tertile 2	Yes	11/52	1.18(0.47–2.95)	1.05(0.41–2.74)
Tertile 3	No	96/456	0.66(0.43–1.02)	0.80(0.53–1.21)
Tertile 3	Yes	10/51	1.02(0.48–2.16)	0.97(0.50–1.90)
Cancer				
Tertile 1	No	36/360	1.00(Reference)	1.00(Reference)
Tertile 1	Yes	11/104	1.81(0.46–7.09)	1.28(0.32–5.16)
Tertile 2	No	23/432	0.32(0.12–0.83)	0.40(0.17–0.95)
Tertile 2	Yes	3/52	0.90(0.18–4.36)	0.80(0.17–3.85)
Tertile 3	No	25/456	0.34(0.15–0.79)	0.42(0.20–0.90)
Tertile 3	Yes	4/51	0.23(0.06–0.92)	0.18(0.05–0.71)
Noncancer				
Tertile 1	No	60/360	1.00(Reference)	1.00(Reference)
Tertile 1	Yes	11/104	1.71(0.92–3.19)	1.16(0.56–2.42)
Tertile 2	No	71/432	1.15(0.71–1.86)	1.38(0.88–2.16)
Tertile 2	Yes	8/52	1.32(0.45–3.84)	1.13(0.34–3.76)
Tertile 3	No	71/456	0.91(0.60–1.38)	1.08(0.71–1.64)
Tertile 3	Yes	6/51	1.69(0.76–3.72)	1.84(0.90–3.77)

a Adjusted for age, sex.

b Adjusted for age, sex, race (Non-Hispanic White, Non-Hispanic Black, Mexican American, others), educational attainment (Less than high school, High school or equivalent, College or above), marital status (Married, Unmarried), Family poverty income ratio, Energy intakes, Caffeine intake.

OBS, oxidative balance scores.

Regarding all-cause mortality, in MV models 1 and 2, within each OBS tertile group, there was no consistent pattern in mortality risk between patients with and without depression. Furthermore, there were no significant differences among the groups.

Regarding cancer mortality, in MV models 1 and 2, within each OBS tertile group, there was no consistent pattern in cancer mortality risk between patients with depression and those without depression. However, in the highest OBS group (tertile 3), the cancer mortality risk of patients with depression was lower than that of those without depression (e.g., in MV model 2, HR for patients with depression = 0.18, 95% CI: 0.05–0.71; HR for patients without depression = 0.42, 95% CI: 0.20–0.90).

Regarding noncancer-related mortality, in MV models 1 and 2, within each OBS tertile group, there was no consistent pattern in noncancer-related mortality risk between patients with depression and those without depression. Moreover, the differences between groups were not statistically significant. These results indicate that in the highest OBS group, the mortality risk of patients with depression was lower, suggesting that depression was a protective factor.

**Figure 4:**
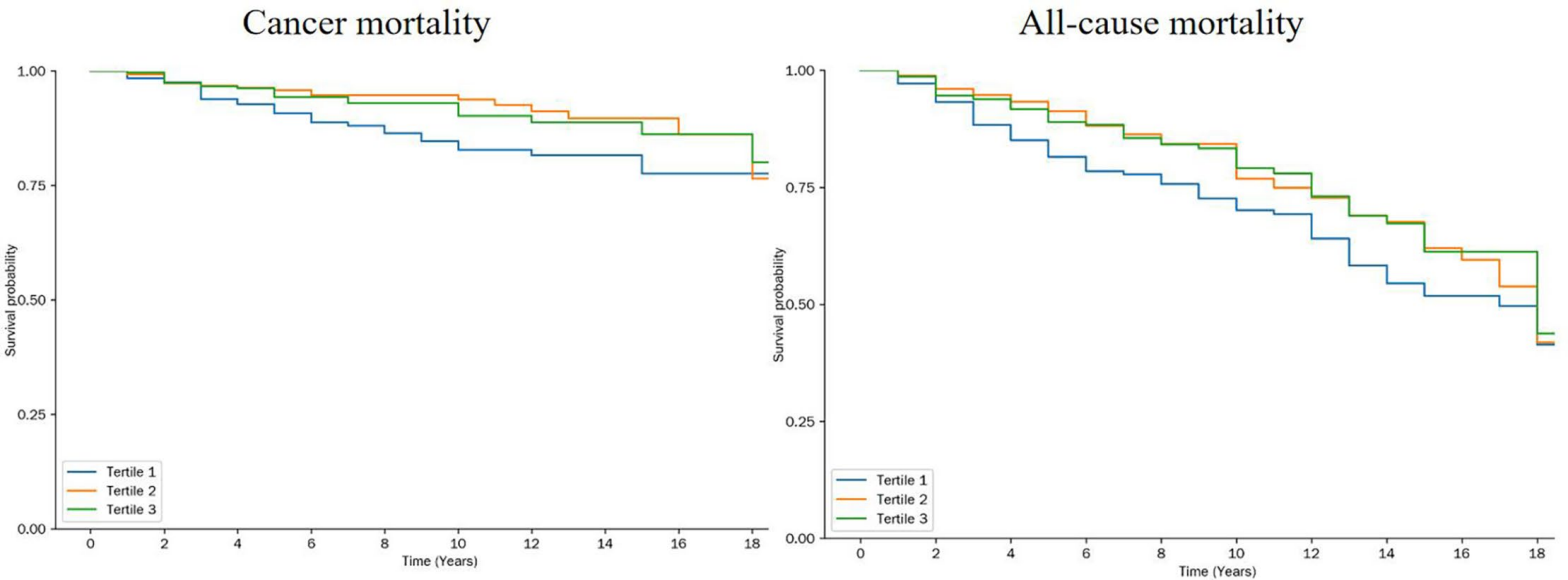
Kaplan Meier for all-cause mortality and cancer mortality by tertiles of the OBS. log-rank-test was used to evaluate differences.

Kaplan–Meier survival analysis revealed that participants in the middle OBS tertile (Tertile 2) had significantly lower cancer-specific mortality compared to that of those in the lowest tertile (Tertile 1) (*p* = 0.023). In contrast, the difference in all-cause mortality across OBS tertiles was less pronounced but statistically significant (*p* = 0.045) (Figure 4). Figure 4 illustrates the survival curves stratified by OBS tertiles, highlighting a protective association between higher OBS and cancer mortality. However, stratified Cox regression analyses did not identify any significant interactions between OBS and covariates with respect to cancer-specific or all-cause mortality (all interaction *p* > 0.05) (Supplementary Table S4). These results suggest that while higher OBS is associated with improved survival outcomes, the magnitude of this effect may not vary substantially across different demographic or clinical subgroups.

Supplementary Table S5 presents the combined effects of lifestyle, dietary OBS, and depression on the mortality of cancer survivors. In terms of all-cause mortality, in most cases, after adjusting for confounding factors, the differences between patients with and without depression within each tertile were not significant. However, the risk of death in the high OBS score group showed a decreasing trend. Depression affected cancer mortality in some groups before adjustment; however, it was not significant after adjustment, and trends remained in specific groups. The situation was similar for noncancer-related mortality, with low risk in the high OBS group and an unremarkable effect of depression. Overall, the results indicated that OBS was related to mortality, depression was affected by multiple factors, and the combined effect of the two was complex and requires further investigation.

## Discussion

### Major findings

This nationally representative analysis highlights the pivotal roles of oxidative balance and depressive symptoms in shaping survival outcomes among adult cancer survivors. Consistent with prior research, we observed a significant inverse association between OBS and cancer-specific mortality, reinforcing the prognostic relevance of redox homeostasis in oncology (20). The OBS, as an integrative metric reflecting both pro- and antioxidant exposures, has been previously linked to reduced risks of cardiometabolic diseases and overall mortality. Our findings extend these associations to cancer survivorship, providing further support for the protective effects of favorable oxidative profiles in this vulnerable population.

Of particular interest, depression alone was not significantly associated with cancer-related death, underscoring the complex and often conflicting nature of psychological comorbidities in cancer prognosis (21). However, in stratified analyses, depressive symptoms were paradoxically associated with lower cancer mortality in individuals with high OBS. This unexpected interaction challenges conventional assumptions and suggests that the effects of depression on survival may be context-dependent, potentially moderated by nutritional and lifestyle-related oxidative balance.

These findings underscore the need for a multidimensional framework in survivorship research, where behavioral, nutritional, and psychological domains are jointly considered. Previous studies (22) have emphasized the role of antioxidant-rich diets in cancer prevention, and Yang et al. (23) demonstrated inverse associations between OBS and systemic inflammation using NHANES data. To the best of our knowledge, our study is among the first to reveal how depressive symptoms may interact with redox status to influence long-term mortality risk. Collectively, the results highlight OBS as a robust biomarker in cancer epidemiology and raise important questions about the bidirectional influences between mental health and oxidative stress in survivorship trajectories.

### Exploration of potential mechanisms

Although this study did not directly assess mechanistic pathways, several plausible biological and behavioral hypotheses may explain the observed interaction between depression, oxidative balance, and cancer mortality. These mechanisms remain speculative but offer important directions for future investigation.

From a biological standpoint, depression is frequently associated with dysregulation of the hypothalamic–pituitary–adrenal (HPA) axis, resulting in elevated circulating cortisol levels (24, 25). Cortisol modulates oxidative homeostasis by influencing key metabolic and inflammatory pathways, and in some contexts, may enhance the activity of antioxidant enzymes such as glutathione peroxidase and superoxide dismutase (26, 27). In cancer cells, glucocorticoids suppress tumor proliferation, invasion, and metastasis by interfering with intracellular signaling cascades (28, 29). Moreover, depression-related reductions in serotonin and other neurotransmitters may alter immune surveillance mechanisms, potentially enhancing antitumor immunity in the presence of high OBS (30, 31).

Behavioral and lifestyle factors may also contribute. While depression is typically linked to maladaptive behaviors, some individuals, especially those with higher health literacy or clinical engagement, may adopt more health-conscious behaviors in response to illness. These may include dietary changes (e.g., reduced intake of saturated fats, increased consumption of antioxidant-rich fruits and vegetables) that elevate OBS (32–34). Additionally, engagement in light-to-moderate physical activity (e.g., walking, yoga) is possible even in individuals with depression, particularly when supported by family or healthcare providers (35, 36). Such activities enhance redox stability, reduce systemic inflammation, and promote immune competence. Increased vigilance and proactive health-seeking behaviors in patients with depression, such as adherence to follow-up care or earlier reporting of symptoms, could also partially mediate improved outcomes (37, 38).

Nonetheless, the finding that depressive symptoms were associated with reduced cancer mortality specifically in the highest OBS tertile remains counterintuitive and warrants cautious interpretation. Reverse causality is a potential explanation, survivors who live longer may be more likely to report depressive symptoms as a result of long-term disease burden. Additionally, residual confounding cannot be ruled out. Important prognostic variables such as cancer stage, treatment modality (e.g., chemotherapy, immunotherapy), and psychosocial support networks were not available in the NHANES dataset and may have influenced both depression status and survival. Lastly, misclassification bias stemming from self-reported depression (based on PHQ-9) may have attenuated or distorted true associations.

Future mechanistic and longitudinal studies, ideally incorporating biological markers (e.g., cortisol, cytokine profiles), treatment history, and validated psychiatric diagnoses, are essential to unravel the interplay between oxidative balance, mental health, and survival trajectories in cancer survivors.

### Clinical significance and implications

The findings of this research hold direct and significant value for the clinical management of cancer. During the risk stratification and prognostic assessment of patients with cancer, clinicians should abandon the previous single-factor assessment model and incorporate OBS and depression status into a comprehensive consideration. For patients with depression and high OBS, traditional treatment strategies need to be re-examined (39). During treatment, it is crucial to closely monitor patients’ psychological states and intensify psychological support and intervention. For example, professional psychological counseling should be provided, psychological treatment courses should be conducted, or patient mutual aid groups should be organized to help patients relieve their depressive emotions and improve their psychological state (40). However, when formulating treatment plans, appropriate adjustments to drug dosages or the selection of drugs with less impact on oxidative stress and psychological state can be considered to avoid aggravating the psychological burden on patients or interfering with the body’s oxidative balance due to treatment. The study results highlight the vital role that interdisciplinary cooperation plays in the comprehensive treatment of cancer. Oncologists should collaborate closely with psychiatrists to jointly develop personalized and comprehensive treatment plans for patients with cancer and improve their survival quality and prognosis.

### Study limitations

Although this study obtained significant findings based on large-sample data from the NHANES database, it still has certain limitations. First, the database was observational. Although many confounding factors were adjusted as much as possible during the analysis, it was difficult to completely rule out the interference of other potential unmeasured or unknown factors. For example, factors such as differences in patients’ genetic backgrounds, exposure levels to environmental pollutants, and socioeconomic status may have affected the results. Second, the measurement of some variables was indirect and contained errors. For example, the calculation of OBS depends on the limited test results of oxidative stress biomarkers in the database, which may not have comprehensively and accurately reflected the body’s oxidative balance state. Although the scale used for depression assessment has been verified, it may still be affected by patients’ subjective factors and cultural background differences, resulting in inaccurate classification of depression status. Third, although the research sample had certain representativeness, the sample size in specific cancer types or population subgroups may have been relatively limited, which may have affected the reliability and extrapolation of the results in these subgroups. Future studies can conduct prospective, multicenter clinical studies, incorporate more comprehensive variable indicators, and use more accurate measurement methods to overcome these limitations and further verify the findings of this study.

### Future research directions

Several key areas will be the focus of future research. First, prospective interventional studies must be conducted. Cancer patients can be categorized into groups and invited to take part in randomized controlled trials. The aim is to investigate the impacts of antioxidants and psychological interventions, either individually or in combination, and to establish causal relationships. Molecular biology techniques can be used to analyze the biological mechanisms and clarify the roles of genes, proteins, and metabolites in this process. Research on specific cancer types, stages, and population subgroups can be refined to obtain targeted results. Multicenter cooperation can be expanded to increase the sample size and enhance reliability. Long-term dynamic monitoring can be performed using wearable devices and other methods. Moreover, prediction models can be established to achieve early warning and personalized intervention, thereby comprehensively promoting research progress in this field.

## Conclusion

This study, using nationally representative NHANES data, demonstrates that both oxidative balance and depression influence cancer-specific mortality. The novel finding that depression may be cancer protective among individuals with high OBS suggests a complex interaction between psychological and nutritional factors in cancer survivorship. However, owing to the observational nature of the study and potential residual confounding, these results should be interpreted with caution. Prospective studies and mechanistic research are needed to clarify causal pathways and validate these associations. Integrating psychological and nutritional assessments into survivorship care may offer new avenues for improving outcomes in patients with cancer.

## Data Availability

Publicly available datasets were analyzed in this study. This data can be found at: https://www.cdc.gov/nchs/nhanes/.
