# Supplementary material for: The relationship between oxidative balance score, depression, and survival among adult cancer survivors in the United States
Source: Front Nutr. 2025 Jul 16;12:1622588. doi: 10.3389/fnut.2025.1622588 (PMC12307194; doi:10.3389/fnut.2025.1622588)
Supplement: Supplementary file 1 [file Table_1.docx]

Table S1. Oxidative balance score assignment scheme.

| OBS component | Property | Male | | | Female | | |
| --- | --- | --- | --- | --- | --- | --- | --- |
|  |  | 0 | 1 | 2 | 0 | 1 | 2 |
| Dietary OBS components |  |  |  |  |  |  |  |
| Calcium(mg/d) | A | <752.00 | 752.00-1034.50 | ≥1034.50 | ＜602.50 | 603.50-926.00 | ≥926.00 |
| Carotene(RE/d) | A | ＜816.00 | 816.00-2704.00 | ≥2704.00 | ＜760.50 | 758.00-2685.00 | ≥2685.00 |
| Copper (mg/d) | A | ＜1.03 | 1.03-1.40 | ≥1.40 | ＜0.84 | 0.84-1.18 | ≥1.18 |
| Total fat (g/d) | A | ＜67.91 | 67.91-95.03 | ≥95.03 | ＜51.38 | 51.38-73.38 | ≥73.38 |
| Dietary fiber (g/d) | A | ＜13.50 | 13.50-20.10 | ≥20.10 | ＜10.65 | 10.65-16.50 | ≥16.50 |
| Iron(mg/d) | A | ＜12.30 | 12.30-17.93 | ≥17.93 | ＜9.37 | 9.37-13.34 | ≥13.34 |
| Magnesium (mg/d) | A | ＜254.50 | 254.50-339.50 | ≥339.50 | ＜198.50 | 198.50-279.50 | ≥279.50 |
| Niacin (mg/d) | A | ＜21.04 | 21.04-28.61 | ≥28.61 | ＜15.40 | 15.40-21.29 | ≥21.29 |
| Riboflavin (mg/d) | A | ＜1.82 | 1.82-2.53 | ≥2.53 | ＜1.38 | 1.38-2.02 | ≥2.02 |
| Selenium(mcg/d) | A | ＜93.70 | 93.70-126.65 | ≥126.65 | ＜70.20 | 70.20-100.00 | ≥100.00 |
| Total folate (mcg/d) | A | ＜316.50 | 316.50-466.00 | ≥466.00 | ＜250.00 | 250.00-353.00 | ≥353.00 |
| Vitamin B6 (mg/d) | A | ＜1.71 | 1.71-2.42 | ≥2.42 | ＜1.24 | 1.24-1.83 | ≥1.83 |
| Vitamin B12 (mcg/d) | A | ＜3.61 | 3.61-5.86 | ≥5.86 | ＜2.50 | 2.50-4.46 | ≥4.46 |
| Vitamin C (mg/d) | A | ＜47.30 | 47.30-97.65 | ≥97.65 | ＜37.10 | 37.10-89.40 | ≥89.40 |
| Vitamin E (ATE) (mg/d) | A | ＜6.15 | 6.15 -9.66 | ≥9.66 | ＜4.93 | 4.93-7.68 | ≥7.68 |
| Zinc (mg/d) | A | ＜9.57 | 9.57-13.48 | ≥13.48 | ＜6.96 | 6.96-10.14 | ≥10.14 |
| Lifestyle OBS components |  |  |  |  |  |  |  |
| Physical  (MET-minute/week) | A | ＜200.00 | 200.00-1840.00 | ≥1840.00 | ＜40.00 | 40.00-1200.00 | ≥1200.00 |
| Cotinine (ng/ml) | P | ＜0.02 | 0.02-0.08 | ≥0.08 | ＜0.02 | 0.02-0.08 | ≥0.08 |
| Alcohol (g/d) | P | ≥30.00 | 0-30.00 | None | ≥15.00 | 0.00-15.00 | None |
| Body mass index (kg/m^2^) | A | ＜25.93 | 25.93-30.20 | ≥30.20 | ＜25.90 | 25.90-32.00 | ≥32.00 |

A stood for the antioxidant, P for the pro-oxidant, RE for the retinal equivalent, ATE for the alpha-tocopherol equivalent, and MET for the metabolic equivalent. Abbreviation and acronyms: OBS, oxidative balance score.

Table S2. The baseline characteristics of females by tertiles of the OBS: National Health and Nutrition Examination Survey 1999–2018 (NHANES 1999–2018)^a^.

| Characteristics | Total (3216) | Tertile 1 | Tertile 2 | Tertile 3 | *P* value |
| --- | --- | --- | --- | --- | --- |
| Age | 60.81(0.68) | 59.45(1.24) | 60.98(1.17) | 61.62(1.11) | 0.0697 |
| Family poverty income ratio | 2.98 (0.08) | 2.35 (0.14) | 3.10 (0.16) | 3.33 (0.14) | <.0001 |
| Energy intakes | 1703.34 (25.82) | 1353.51 (32.13) | 1629.34 (44.33) | 2015.13 (36.98) | <.0001 |
| Caffeine intakes | 169.20 (7.24) | 184.88 (18.68) | 161.41 (12.54) | 164.71 (9.76) | 0.2584 |
| Marital status, Married (n, %) |  |  |  |  | 0.0370 |
| Married | 439 (56.96) | 130 (47.53) | 148 (62.96) | 154 (58.54) |  |
| Unmarried | 419 (43.04) | 148 (52.47) | 130 (37.04) | 141 (41.46) |  |
| Educational level (n, %) |  |  |  |  | <.0001 |
| College or above | 492 (68.05) | 113 (48.71) | 172 (71.98) | 207 (78.43) |  |
| High school or equivalent | 186 (20.58) | 75 (29.55) | 51 (17.59) | 60 (16.76) |  |
| Less than high school | 173 (11.37) | 90 (21.74) | 55 (10.43) | 28 (4.80) |  |
| Race (n, %) |  |  |  |  | 0.0675 |
| Non-Hispanic White | 575 (84.71) | 168 (79.91) | 184 (83.13) | 223 (89.47) |  |
| Non-Hispanic Black | 100 (4.89) | 47 (7.39) | 31 (4.87) | 22 (3.13) |  |
| Mexian American | 68 (2.76) | 29 (4.03) | 23 (2.43) | 16 (2.14) |  |
| Others | 108 (7.65) | 34 (8.68) | 40 (9.57) | 34 (5.26) |  |
| History of comorbidities, no (n, %) |  |  |  |  | 0.0629 |
| No | 283 (40.35) | 69 (31.76) | 95 (41.99) | 119 (45.05) |  |
| Yes | 568 (59.65) | 209 (68.24) | 183 (58.01) | 176 (54.95) |  |
| Antidepressant(n, %) |  |  |  |  |  |
| No | 784 (89.78) | 247 (84.87) | 263 (91.33) | 274 (91.93) | 0.1939 |
| Yes | 67 (10.22) | 31 (15.13) | 15 (8.67) | 21 (8.07) |  |

^a^ All estimates accounted for complex survey designs in NHANES. Values were mean ± standard error for continuous variables and numbers (percentages) for categorical variables. Abbreviation and acronyms: OBS, oxidative balance score; PIR, family income-to-poverty ratio.

Table S3: The baseline characteristics of males by tertiles of the OBS: National Health and Nutrition Examination Survey 1999–2018 (NHANES 1999–2018)^a^.

| Characteristics | Total (604) | Tertile 1 | Tertile 2 | Tertile 3 | *P* value |
| --- | --- | --- | --- | --- | --- |
| Age | 64.52 (0.84) | 63.21 (1.74) | 64.69(1.36) | 65.41(1.40) | 0.6329 |
| Family poverty income ratio | 3.49 (0.10) | 2.92 (0.19) | 3.60 (0.17) | 3.84 (0.14) | <.0001 |
| Energy intakes | 2220.68 (42.94) | 1801.01 (65.56) | 2115.00 (61.41) | 2626.65 (56.42) | <.0001 |
| Caffeine intakes | 186.57 (9.41) | 171.56 (17.88) | 187.03 (18.21) | 197.88 (13.80) | 0.5483 |
| Marital status, Married (n, %) |  |  |  |  | 0.0411 |
| Married | 414 (74.30) | 130 (64.74) | 137 (76.84) | 147 (79.81) |  |
| Unmarried | 190 (35.70) | 78 (35.26) | 47 (23.16) | 65 (20.19) |  |
| Educational level (n, %) |  |  |  |  | 0.4586 |
| College or above | 347 (69.29) | 98 (63.25) | 114 (70.69) | 135 (72.91) |  |
| High school or equivalent | 157 (22.73) | 60 (26.10) | 43 (21.10) | 54 (21.33) |  |
| Less than high school | 100 (7.99) | 50 (10.64) | 27 (8.20) | 23 (5.76) |  |
| Race (n, %) |  |  |  |  | 0.2815 |
| Non-Hispanic White | 445 (91.83) | 143 (85.86) | 137 (87.43) | 165 (92.15) |  |
| Non-Hispanic Black | 90 (5.88) | 37 (5.85) | 25 (5.26) | 28 (3.20) |  |
| Mexian American | 22 (2.89) | 9 (1.87) | 10 (2.91) | 3 (0.68) |  |
| Others | 47 (7.22) | 19 (6.42) | 12 (4.41) | 16 (3.97) |  |
| History of comorbidities, no (n, %) |  |  |  |  | 0.2122 |
| No | 150 (34.89) | 44 (23.77) | 40 (26.99) | 66 (36.19) |  |
| Yes | 454 (75.62) | 164 (76.23) | 144 (73.01) | 146 (63.82) |  |
| Antidepressant(n, %) |  |  |  |  | 0.4113 |
| No | 575 (93.23) | 196 (91.76) | 177 (91.63) | 202 (95.57) |  |
| Yes | 69 (6.77) | 12 (8.24) | 7 (8.37) | 10 (4.43) |  |

^a^ All estimates accounted for complex survey designs in NHANES. Values were mean ± standard error for continuous variables and numbers (percentages) for categorical variables. Abbreviation and acronyms: OBS, oxidative balance score; PIR family income-to-poverty ratio.

Table S4. Stratified analysis of associations of OBS with all-cause mortality among US cancer survivors aged 20 years or older, NHANES 1999–2018^a^.

| Stratified factors |  | cancer mortality (OR 95%CI) | *P* for interaction |
| --- | --- | --- | --- |
| Cancer mortality  **Sex** |  |  | 0.2305 |
|  | Tertile 1 | 1.00 (reference) |  |
| Male | Tertile 2 | 1.31 (0.51 – 3.37) |  |
|  | Tertile 3 | 0.87 (0.35 – 2.20) |  |
|  | Tertile 1 | 1.00 (reference) |  |
| Female | Tertile 2 | 0.65 (0.28 - 1.50) |  |
|  | Tertile 3 | 0.58 (0.22 - 1.50) |  |
| **Age (years)** |  |  | 0.5210 |
|  | Tertile 1 | 1.00 (reference) |  |
| Age < 50 | Tertile 2 | 0.42 (0.08 - 2.15) |  |
|  | Tertile 3 | 3.55 (1.13 - 11.16) |  |
|  | Tertile 1 | 1.00 (reference) |  |
| Age ≥ 50 | Tertile 2 | 1.03 (0.54 - 1.95) |  |
|  | Tertile 3 | 0.79 (0.41 - 1.52) |  |
| **Race** |  |  | 0.1664 |
|  | Tertile 1 | 1.00 (reference) |  |
| Non-Hispanic White | Tertile 2 | 0.80 (0.40 - 1.58) |  |
|  | Tertile 3 | 1.52 (0.62 - 3.78) |  |
|  | Tertile 1 | 1.00 (reference) |  |
| Others | Tertile 2 | 1.42 (0.69 - 2.93) |  |
|  | Tertile 3 | 1.64 (0.44 - 6.13) |  |
| **Education** |  |  | 0.2372 |
|  | Tertile 1 | 1.00 (reference) |  |
| College or above | Tertile 2 | 0.50 (0.16 - 1.52) |  |
|  | Tertile 3 | 0.59 (0.25 - 1.40) |  |
|  | Tertile 1 | 1.00 (reference) |  |
| Others | Tertile 2 | 1.03 (0.49 - 2.17) |  |
|  | Tertile 3 | 0.75 (0.34 - 1.64) |  |
| **Marriage** |  |  | 0.7597 |
|  | Tertile 1 | 1.00 (reference) |  |
| Married | Tertile 2 | 1.09 (0.48 - 2.50) |  |
|  | Tertile 3 | 0.85 (0.36 - 1.98) |  |
|  | Tertile 1 | 1.00 (reference) |  |
| Unmarried | Tertile 2 | 0.78 (0.33 - 1.84) |  |
|  | Tertile 3 | 0.60 (0.21 - 1.73) |  |
| **PIR** |  |  | 0.8641 |
|  | Tertile 1 | 1.00 (reference) |  |
| Low | Tertile 2 | 0.84 (0.43 - 1.64) |  |
|  | Tertile 3 | 0.54 (0.24 - 1.22 ) |  |
|  | Tertile 1 | 1.00 (reference) |  |
| High | Tertile 2 | 0.90 (0.47 - 1.73) |  |
|  | Tertile 3 | 0.65 (0.30 - 1.41) |  |
| All-cause mortality |  |  |  |
| **Sex** |  |  | 0.7022 |
|  | Tertile 1 | 1.00 (reference) |  |
| Male | Tertile 2 | 0.24 (0.07 - 0.86) |  |
|  | Tertile 3 | 0.17 (0.04 - 0.77) |  |
|  | Tertile 1 | 1.00 (reference) |  |
| Female | Tertile 2 | 0.45 (0.17 - 1.14) |  |
|  | Tertile 3 | 0.36 (0.10 - 1.37) |  |
| **Age (years)** |  |  | 0.2318 |
|  | Tertile 1 | 1.00 (reference) |  |
| Age < 50 | Tertile 2 | < 0.001 |  |
|  | Tertile 3 | 4.30 (1.05 - 17.64) |  |
|  | Tertile 1 | 1.00 (reference) |  |
| Age ≥ 50 | Tertile 2 | 0.33 (0.13 - 0.84) |  |
|  | Tertile 3 | 0.19 (0.07 - 0.57) |  |
| **Race** |  |  | 0.3530 |
|  | Tertile 1 | 1.00 (reference) |  |
| Non-Hispanic White | Tertile 2 | 0.48 (0.18 - 1.31) |  |
|  | Tertile 3 | 0.76 (0.27 - 2.13) |  |
|  | Tertile 1 | 1.00 (reference) |  |
| Others | Tertile 2 | 0.30 (0.11 – 0.81) |  |
|  | Tertile 3 | 0.19 (0.06 – 0.61) |  |
| **Education** |  |  | 0.6608 |
|  | Tertile 1 | 1.00 (reference) |  |
| College or above | Tertile 2 | 0.29 (0.11 - 0.77) |  |
|  | Tertile 3 | 0.23 (0.08 - 0.62) |  |
|  | Tertile 1 | 1.00 (reference) |  |
| Others | Tertile 2 | 0.65 (0.18 - 2.31) |  |
|  | Tertile 3 | 0.32 (0.06 - 1.85) |  |
| **Marriage** |  |  | 0.2605 |
|  | Tertile 1 | 1.00 (reference) |  |
| Married | Tertile 2 | 0.45 (0.17 - 1.19) |  |
|  | Tertile 3 | 0.33 (0.10 - 1.13) |  |
|  | Tertile 1 | 1.00 (reference) |  |
| Unmarried | Tertile 2 | 0.25 (0.07 – 0.86) |  |
|  | Tertile 3 | 0.17 (0.05 – 0.65) |  |
| **PIR** |  |  | 0.0696 |
|  | Tertile 1 | 1.00 (reference) |  |
| Low | Tertile 2 | 0.28 (0.11 - 0.75) |  |
|  | Tertile 3 | 0.11 (0.04 - 0.33) |  |
|  | Tertile 1 | 1.00 (reference) |  |
| High | Tertile 2 | 0.42 (0.13 - 1.38) |  |
|  | Tertile 3 | 0.39 (0.10 - 1.51) |  |

^a^Adjusted for confounding factors such as age, gender, race, education, poverty–income ratio.

Abbreviation and acronyms: PIR family income-to-poverty ratio. OBS, oxidative balance scores

Table S5. Joint association of lifestyle/ dietary OBS and depression status with all-cause, cancer, and noncancer mortality among US cancer survivors aged 20 years or older, National Health and Nutrition Examination Survey 1999-2018

| Mortality outcome | Depression | Death/No | MV model 1^a^ | MV model 2^b^ |
| --- | --- | --- | --- | --- |
| Lifestyle OBS  All Cause |  |  |  |  |
| Tertile 1 | No | 133/503 | 1.00(Reference) | 1.00(Reference) |
| Tertile 1 | Yes | 21/120 | 1.24(0.66-2.33) | 0.93(0.52-1.67) |
| Tertile 2 | No | 59/291 | 0.64(0.33-1.24) | 0.69(0.35-1.36) |
| Tertile 2 | Yes | 11/40 | 1.28(0.62-2.62) | 1.05(0.51-2.17) |
| Tertile 3 | No | 94/454 | 0.54(0.37-0.79) | 0.66(0.43-1.00) |
| Tertile 3 | Yes | 11/47 | 1.33(0.54-3.29) | 1.23(0.57-2.63) |
| Cancer |  |  |  |  |
| Tertile 1 | No | 40/503 | 1.00(Reference) | 1.00(Reference) |
| Tertile 1 | Yes | 8/120 | 2.85(1.07-7.60) | 1.91(0.71-5.09) |
| Tertile 2 | No | 18/291 | 1.43(0.45-4.55) | 1.63(0.54-4.94) |
| Tertile 2 | Yes | 5/40 | 0.98(0.31-3.12) | 0.70(0.24-2.09) |
| Tertile 3 | No | 26/454 | 0.79(0.46-1.37) | 1.05(0.53-2.07) |
| Tertile 3 | Yes | 5/47 | 1.34(0.39-4.63) | 1.02(0.28-3.76) |
| Noncancer |  |  |  |  |
| Tertile 1 | No | 93/503 | 1.00(Reference) | 1.00(Reference) |
| Tertile 1 | Yes | 13/120 | 0.74(0.36-1.49) | 0.60(0.29-1.25) |
| Tertile 2 | No | 41/291 | 0.45(0.26-0.80) | 0.46(0.26-0.83) |
| Tertile 2 | Yes | 6/40 | 1.34(0.55-3.30) | 1.17(0.48-2.83) |
| Tertile 3 | No | 68/454 | 0.47(0.30-0.75) | 0.55(0.34-0.89) |
| Tertile 3 | Yes | 6/47 | 1.38(0.42-4.55) | 1.42(0.51-3.98) |
| Dietary OBS |  |  |  |  |
| All Cause |  |  |  |  |
| Tertile 1 | No | 101/401 | 1.00(Reference) | 1.00(Reference) |
| Tertile 1 | Yes | 22/105 | 2.24(1.09-4.61) | 1.48(0.70-3.16) |
| Tertile 2 | No | 91/402 | 0.95(0.61-1.48) | 1.09(0.72-1.65) |
| Tertile 2 | Yes | 12/46 | 2.09(0.85-5.13) | 1.67(0.65-4.29) |
| Tertile 3 | No | 94/445 | 0.74(0.48-1.14) | 0.86(0.56-1.30) |
| Tertile 3 | Yes | 9/56 | 0.71(0.35-1.44) | 0.65(0.32-1.34) |
| Cancer |  |  |  |  |
| Tertile 1 | No | 36/401 | 1.00(Reference) | 1.00(Reference) |
| Tertile 1 | Yes | 11/105 | 2.06(0.52-8.08) | 1.38(0.34-5.66) |
| Tertile 2 | No | 19/402 | 0.26(0.10-0.70) | 0.32(0.14-0.77) |
| Tertile 2 | Yes | 3/46 | 0.97(0.19-5.00) | 0.81(0.17-3.86) |
| Tertile 3 | No | 29/445 | 0.48(0.22-1.07) | 0.61(0.31-1.19) |
| Tertile 3 | Yes | 4/56 | 0.27(0.07-1.02) | 0.22(0.06-0.82) |
| Noncancer |  |  |  |  |
| Tertile 1 | No | 65/401 | 1.00(Reference) | 1.00(Reference) |
| Tertile 1 | Yes | 11/105 | 1.92(1.04-3.53) | 1.29(0.62-2.69) |
| Tertile 2 | No | 72/402 | 1.49(0.93-2.38) | 1.63(1.03-2.60) |
| Tertile 2 | Yes | 9/46 | 3.05(1.10-8.46) | 2.40(0.74-7.78) |
| Tertile 3 | No | 65/445 | 0.92(0.59-1.42) | 1.02(0.65-1.60) |
| Tertile 3 | Yes | 5/56 | 1.04(0.46-2.37) | 1.04(0.43-2.51 |

^a^Adjusted for age, sex.
^b^Adjusted for age, sex, race(Non-Hispanic White, Non-Hispanic Black, Mexian American, others), educational attainment (Less than high school, High school or equivalent,College or above), marital status (Married, Unarried),Family poverty income ratio,Energy intakes,Caffeine intakes

Abbreviation and acronyms: OBS, oxidative balance scores
