# Supplementary figures and images for: The relationship between oxidative balance score, depression, and survival among adult cancer survivors in the United States
Source: Front Nutr. 2025 Jul 16;12:1622588. doi: 10.3389/fnut.2025.1622588 (PMC12307194; doi:10.3389/fnut.2025.1622588)

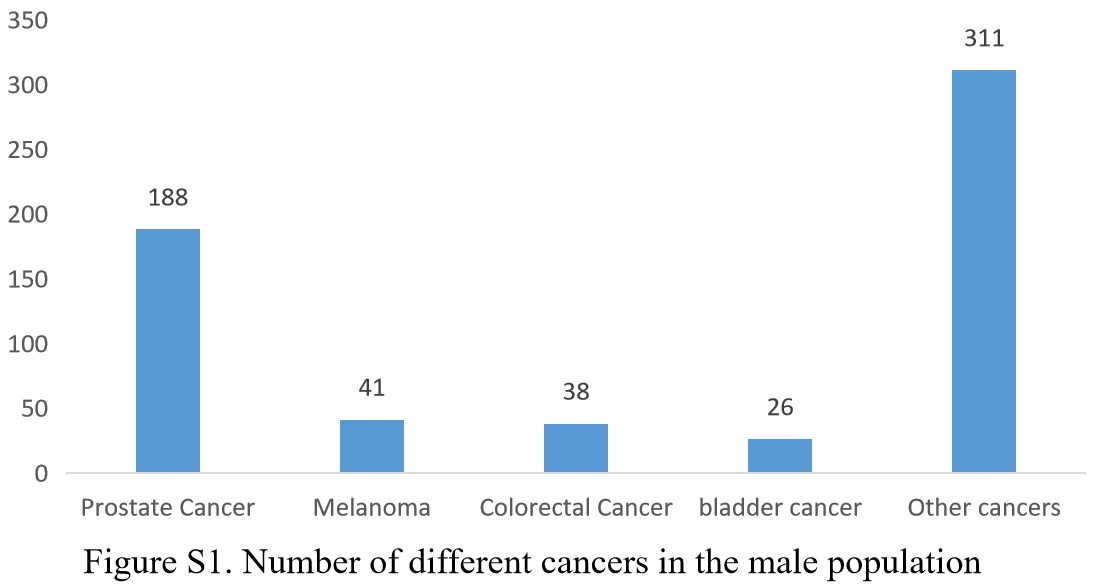

Supplement: Supplementary file 2 [file Image_1.jpeg]

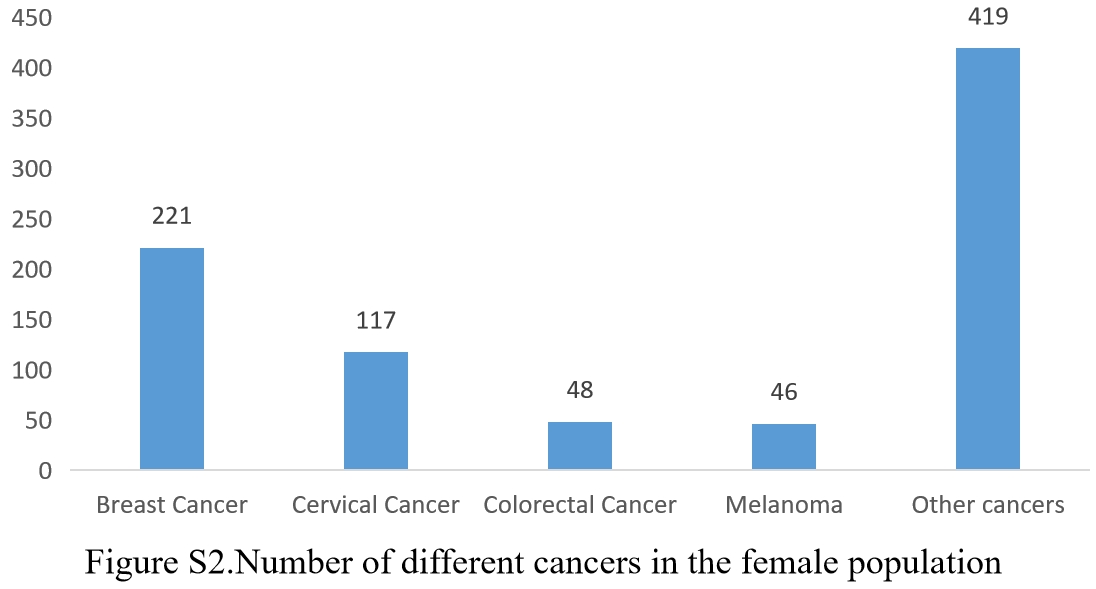

Supplement: Supplementary file 3 [file Image_2.jpeg]
